# Are In Vitro Human Blood–Brain–Tumor-Barriers Suitable Replacements for In Vivo Models of Brain Permeability for Novel Therapeutics?

**DOI:** 10.3390/cancers13050955

**Published:** 2021-02-25

**Authors:** Archana Prashanth, Heather Donaghy, Shihani P. Stoner, Amanda L. Hudson, Helen R. Wheeler, Connie I. Diakos, Viive M. Howell, Georges E. Grau, Kelly J. McKelvey

**Affiliations:** 1 Bill Walsh Translational Cancer Research Laboratory, Kolling Institute, Faculty of Medicine and Health, The University of Sydney, St Leonards, NSW 2065, Australia; apra3545@uni.sydney.edu.au (A.P.); heather.donaghy@sydney.edu.au (H.D.); shihani.stoner@sydney.edu.au (S.P.S.); amanda.hudson@sydney.edu.au (A.L.H.); helen.wheeler@health.nsw.gov.au (H.R.W.); connie.diakos@sydney.edu.au (C.I.D.); viive.howell@sydney.edu.au (V.M.H.); 2 Department of Medical Oncology, Northern Sydney Cancer Centre, Royal North Shore Hospital, St Leonards, NSW 2065, Australia; 3 Vascular Immunology, Department of Pathology, School of Pathology, Faculty of Medicine and Health, The University of Sydney, Camperdown, NSW 2050, Australia; georges.grau@sydney.edu.au

**Keywords:** blood brain barrier, permeability, neurovascular unit, brain microvascular endothelial cells, induced pluripotent stem cells, tight junctions, transendothelial electrical resistance, brain cancer, glioblastoma, diffuse intrinsic pontine glioma

## Abstract

**Simple Summary:**

Brain cancers are a devastating disease with no cure. The aim of the study was to determine whether in vitro models can replace in vivo models to assess the brain permeability of novel drugs for brain cancer. Using the Preferred Reporting Items for Systematic Reviews and Meta-Analyses guidelines, our systematic review reveals that microfluidic-based in vitro models comprising stem cell-derived endothelial cells, and primary astrocytes, pericytes and neurons can, in part, replicate the physiological ability of in vivo models to mimic patient permeability data. This information will guide the development and use of in vitro models for novel therapeutics of unknown permeability for brain cancer.

**Abstract:**

Background: High grade gliomas (HGG) are incapacitating and prematurely fatal diseases. To overcome the poor prognosis, novel therapies must overcome the selective and restricted permeability of the blood–brain barrier (BBB). This study critically evaluated whether in vitro human normal BBB and tumor BBB (BBTB) are suitable alternatives to “gold standard” in vivo models to determine brain permeability. Methods: A systematic review utilizing the PRISMA guidelines used English and full-text articles from the past 5 years in the PubMed, Embase, Medline and Scopus databases. Experimental studies employing human cell lines were included. Results: Of 1335 articles, the search identified 24 articles for evaluation after duplicates were removed. Eight in vitro and five in vivo models were identified with the advantages and disadvantages compared within and between models, and against patient clinical data where available. The greatest in vitro barrier integrity and stability, comparable to in vivo and clinical permeability data, were achieved in the presence of all cell types of the neurovascular unit: endothelial cells, astrocytes/glioma cells, pericytes and neurons. Conclusions: In vitro co-culture BBB models utilizing stem cell-derived or primary cells are a suitable proxy for brain permeability studies in order to reduce animal use in medical research.

## 1. Introduction

Malignant tumors of the brain and central nervous system (CNS) are among the most rapidly lethal types of cancer [1,2]. Patients diagnosed with high grade gliomas (HGG) have a poor median survival of 14–21 months for adult glioblastoma (GBM) [3] and 4–17 months for pediatric diffuse midline glioma (previously called diffuse intrinsic pontine glioma, DIPG) [4]. Limited advancements in therapeutic options mean that survival rates for GBM and DIPG patients have remained largely unchanged over the last 30 years [5]. To overcome the poor prognosis and survival rates of HGG, the development of novel anti-cancer drug therapies for brain cancer treatment is a major focus of current research. However, the highly selective and restricted permeability of the blood–brain barrier (BBB) is a major constraint to overcome before therapeutic efficacy can be considered.

**Figure 1:**
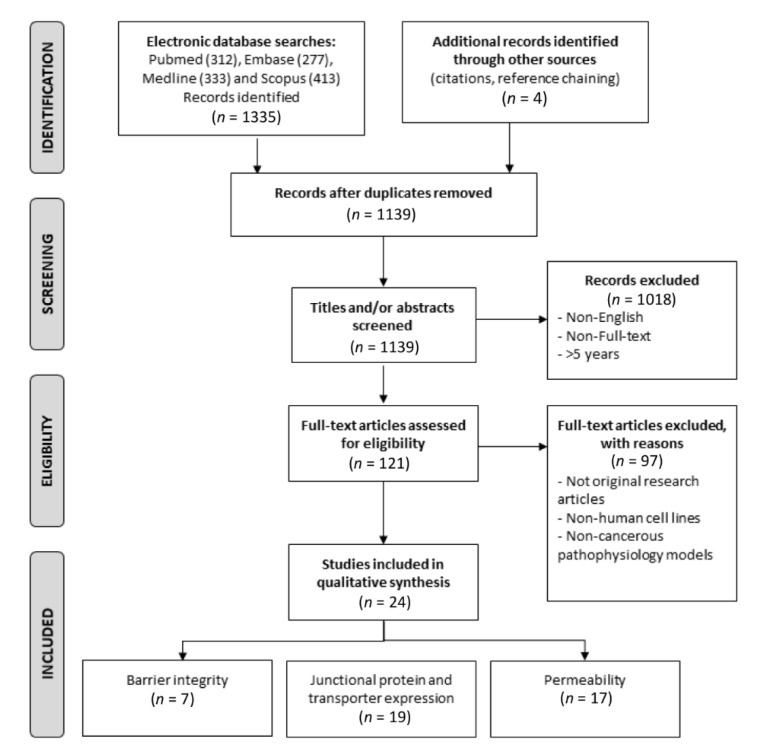
PRIMSA flowchart of the study selection process methodology for the systematic review. Keyword combinations used in the electronic database searches are outlined in Table S2. Figure adapted from Moher and colleagues [14].

The BBB is a selective barrier formed by neurovascular units (NVU), consisting of an endothelial cell (EC) monolayer interacting with astrocyte end-feet [6,7], pericytes, neurons, interneurons, microglia and other immune cells [8] (Figure 1). At interendothelial clefts, tight and adherens junctions bond ECs to each other forming the restrictive barrier function [9] (Figure 1). Constant crosstalk and interaction between cells of the NVU contribute to the structural and signaling-based regulation of transcellular and paracellular transport [10], which determines BBB permeability, and the overall regulation of cerebral circulation [11]. Tumor expansion leads to changed and new properties of the NVU resulting in a more permeable (“leakier”) BBTB with increased fenestrations compared to the BBB [6,10]. However, studies show that rather than being completely disrupted, the BBTB displays heterogenous barrier permeability [10,12]; the peripheral region of the tumor and surrounding parenchyma maintain an intact BBB, but the tumor core may become disrupted and express a more permeable BBB due to regions of hypoxia-induced cell death, a form of necrosis [10]. The regions of comparatively intact BBB at the tumor periphery result in the BBTB retaining efflux (brain-to-blood) transporter protein expression and function [2]. Thus, despite regions of BBB disruption, many anti-cancer therapeutics are still unable to accumulate in the brain parenchyma at concentrations high enough to maintain tumor control—either due to reduced permeability or increased efflux.

Current testing of novel therapeutics relies heavily on small in vivo animal models, particularly rodents [13]. The number of drugs and combinations that can be tested is constrained by ethical, experimental and financial considerations. Thus, identifying an accurate, scalable and high throughput model system that can provide valuable information regarding drug permeability into the brain will be highly beneficial in improving the translation of preclinical success rates.

In this systematic review, our aim was to critically evaluate whether in vitro BBB models can replicate in vivo and clinical data. The in vitro studies were assessed for their ability to replicate barrier integrity, protein and transporter expression and permeability similar to in vivo small animal and human BBB/BBTBs. Models comprising iPSC-ECs and primary astrocytes/glioma cells, pericytes (and neurons), were revealed to be the most suitable replacements for current in vivo models for the purpose of assessing permeability.

## 2. Materials and Methods

The systematic review was performed in accordance with the PRISMA guidelines [14]. The PRISMA Protocols checklist is provided in Table S1.

Published studies were identified using a combination of Medical Subject Headings and free-text terms (Table S1) in the online databases PubMed, Embase, Medline and Scopus. Only English language, full-text articles were included. Where the number of identified articles for a term combination exceeded one hundred, searches were limited by date of publication to the last five years (August 2015 to August 2020). Identified articles were downloaded into an EndNote X9.3.1 for Mac (Clarivate Analytics, Philadelphia, PA, USA) library and duplicate articles removed before the study selection, exclusion and eligibility criteria were applied (Figure 1).

The following data were registered: What in vitro models are currently in use and their advantages and disadvantages? What in vivo models are currently in use and their advantages and disadvantages? How well does in vitro (i) BBB integrity, (ii) junctional and adherens protein and transporter expression, and (iii) permeability match in vivo and available clinical data?

A systematic synthesis of all relevant study results was reported in text, figures and tables to provide an interpretation of all significant study findings.

### Caveat

A key limitation and bias of this systematic review was that data in some identified articles were graphically illustrated and the mean +/− SD data were not reported in the text or supplementary data. This limited the number of studies from which raw data could be acquired and compared. Equally, the numerous combinations of models, EC cell origins, co-culturing and fluorescent tracers and drugs assessed mean that a meta-analysis of the TEER and permeability data was not permissible. It is important to note that the scope of studies analyzed had limited assessment of spheroidal, hollow-fiber, fiber-free and hydrogel scaffold models (1–2 studies each); therefore, only tentative conclusions of their suitability as replacements for animal models can be provided in this review.

## 3. Results

The systematic review was performed according to the Preferred Reporting Items for Systematic Reviews and Meta-Analyses (PRISMA) guidelines. A PRISMA flow chart of the publications is presented in Figure 1. A database search of Pubmed, Medline, Embase and Scopus identified 1335 citations in total. A summary of the keywords used and publications identified is presented in Table S2. After the removal of duplicates, 1139 remained. Following the evaluation of the publication titles and abstracts, 1016 articles were discarded, as the articles did not address the topic of BBB/BBTB models. One article was discarded, as it had been retracted from publication. The full text of the remaining 121 articles was examined for eligibility against the criteria outlined in Figure 1. Of these, 97 articles did not meet the inclusion criteria. In total, 24 articles met the criteria for inclusion in the systematic review.

### 3.1. In Vitro BBB/BBTB Models

**Figure 2:**
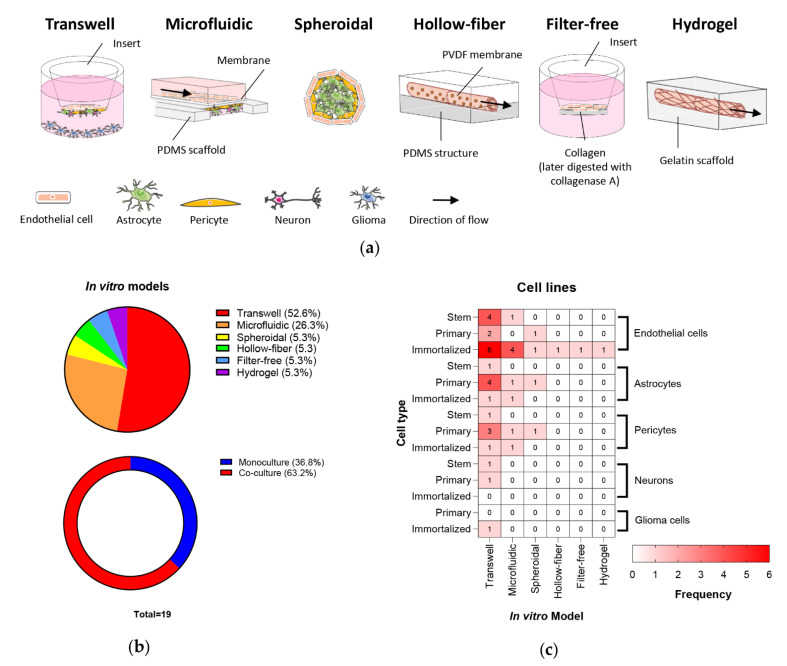
In vitro BBB/BBTB models. (**a**) Schematic representations of transwell, microfluidic, spheroidal, hollow-fiber, filter-free and hydrogel scaffold models. (**b**) Pie and donut chart of the articles employing each in vitro model and the division of EC monoculture and co-culture studies, respectively, and (**c**) a heat map of the utilization of stem, primary and immortalized EC, astrocyte, pericyte, neuron and glioma cell lines. Figure prepared using Servier Medical Art (https://smart.servier.com/ accessed: 3rd November 2020). PVDF, polyvinylidene fluoride; PDMS, polydimethylsiloxane.

Multiple human in vitro BBB models are currently being utilized for BBB research as depicted in Figure 2a. Of the 24 reviewed studies, 19 reported in vitro BBB models: 10 (52.6%) used the transwell system (also called a Boyden chamber assay), five (26.3%) a microfluidic system, one (5.3%) spheroid, one (5.3%) hollow-fiber, one (5.3%) filter-free, and one (5.3%) hydrogel scaffold model (Figure 2b). The filter-free model is a modification of the transwell model, with ECs grown on a collagen matrix that is then digested by collagenase A to create a “filter free” cell layer for subsequent analyses [15]. The hollow-fiber model is a modification of the microfluidic platform utilizing a hollow tube of porous polyvinylidene fluoride membrane encased in a polydimethylsiloxane structure [16]. Of the studies assessed, seven (36.8%) were performed using monocultures, while 12 (63.2%) used 2 to 4 cell co-culture models (Figure 2b).

The utilization of stem, primary and immortalized ECs, astrocyte, pericyte, neurons and glioma cell lines ranged across the platforms (Figure 2c). Immortalized ECs were the most frequently used (14/19 studies; 73.7%), followed by primary astrocytes (6/19; 31.6%) and primary pericytes together with iPSC-ECs (5/19; 26.3%) (Figure 2c and Table S3). The most frequently utilized platform was the transwell with immortalized ECs, namely, hCMEC/D3. The newer platforms—hollow-fiber, filter-free and hydrogel scaffold—all utilized immortalized ECs alone (Figure 2c), likely attributable to the early development of these models. Surprisingly, few studies included glioma cells in the in vitro BBB model. 

Of the two primarily used models, transwells are cost-effective, simple models where the types of NVU cells can be readily employed; however, this model is static and lacks an extracellular or tumor microenvironment. Microfluidic systems enable the assessment of permeability under the physiologically relevant conditions of flow and shear stress, though require purchase of a commercially available device or 3D printing capabilities. However, it should be noted that current microfluidic platforms have some “design/manufacturing” limitations, including membrane thicknesses (10 µm) significantly greater than the physiological basement membrane reducing cell–cell contact, and “vessel” dimeters substantially larger that brain microvascular capillaries (6–9 µm). A summary of the advantages and disadvantages of the various in vitro models is provided in Table 1. 

### 3.2. In Vivo BBB/BBTB Models

In vivo models are the “gold standard” for the investigation of BBB permeability for novel therapeutics due to the presence of BBB architecture; physiological flow/shear stress conditions; and ability to assess pharmacokinetic factors, such as drug metabolism and clearance. Five in vivo models are commonly employed for the assessment of BBB permeability in vivo (Table 2): (i) single and internal carotid artery perfusion, (ii) intravenous tail and cannulated femoral vein injection, (ii) intracerebral or cerebral open flow microdialysis, (iv) primary cell-derived xenografts and (v) genetically engineered mouse models (reviewed by Sharma and colleagues [38]). These models range in their level of technical complexity and pharmacokinetic influence (metabolism/clearance; Table 2). Unlike in vitro models where BBB integrity and permeability can be readily assessed, in vivo models require additional techniques to quantitate drug concentration, requiring additional time, resources and technical proficiency (Table 2), e.g., live imaging (e.g., magnetic resonance imaging and positron emission tomography), or the harvesting of brain tissue or cerebrospinal/brain extracellular fluid followed by liquid chromatography or mass spectrophotometry techniques (e.g., HPLC, UPLC, LC-MS and ICP-MS) [38]. The ability to use in vitro models as an alternate cost and time-effective, high-throughput workflow to accurately predict physiological BBB permeability would be highly beneficial.

### 3.3. How Well Do In Vitro BBB/BBTB Mimic In Vivo and Clinical Physiology?

Several key factors are involved in the determination of the successful replication of the in vivo and patient BBB/BBTB architecture by an in vitro model. This includes (i) the BBB architecture, (ii) barrier integrity, (iii) junctional, adherens and transporter protein expression and (iv) permeability.

#### 3.3.1. BBB Architecture

The NVU comprises the key cell types of the BBB: ECs, astrocytes, pericytes, neurons and immune cells. Within microfluidic models, EC cells form confluent monocultures with complete lumens [18]. However, EC monocultures can cause vessels to fuse, forming elliptical lumens, and vascular network degradation and regression after 7 days [17]. Campisi and colleagues generated microfluidic BBB models of tri-culture with ECs, astrocytes and pericytes. These cultures formed vessel lumens with smaller and more circular cross-sections, and stable, shorter mean vascular branch length—more reflective of in situ vessels, when compared to monocultures and co-cultures (Table 3) [17].

As the blood flows (perfused) along the vessels, it imparts a force to molecules (or cells) trying to pass perpendicular through the endothelial wall (termed shear stress; 1 dyne = 1 g*cm/s^2^). As a result, perfusion and shear stress influence BBB permeability. In BBB vasculature, capillaries are 7–10 µm in diameter, whilst arterioles and venules are 10–90 µm [17]. Brain interstitial flow rate is <500 µL/min [39]. Physiological shear stresses of 10–20 dynes/cm^2^, 4–30 dynes/cm^2^ and 1–4 dynes/cm^2^ have been detected in capillaries, arterioles and venules, respectively [40]. Wevers and colleagues used a microfluidic model with 400 × 220 µm^2^ to replicate in vivo fluid flow and shear stress (~1.2 dynes/cm^2^) [34]. However, the shear stress established in this model is low compared to the aforementioned physiological values. Moya and colleagues tested a hCMEC/D3 hollow-fiber model of 43.5 × 0.7 mm^2^ under continuous flow and shear stress conditions of 0, 94.7, 380 and 2100 µL/min corresponding to 0, 0.77, 3, and 17 dynes/cm^2^, respectively [16]. At 3 dynes/cm^2^, ECs showed the most abundant growth and a 5-fold increase in nitric oxide, an important vascular tone regulator, when compared to static conditions (0 dynes/cm^2^) [16].

#### 3.3.2. Barrier Integrity

Validation of in vitro BBB/BBTB model barrier integrity is necessary to determine if the model exhibits functional physiological characteristics of the barrier akin to in vivo models. Transendothelial electrical resistance (TEER) analysis is a non-invasive method used to determine the barrier integrity of BBB models via quantitative measurement of the electrical resistance across the barrier. A nominal TEER value of ≥500 Ω/cm^2^ has been proposed to signify an intact BBB [22], where values above this indicate physiologically relevant BBB barriers.

**Figure 3:**
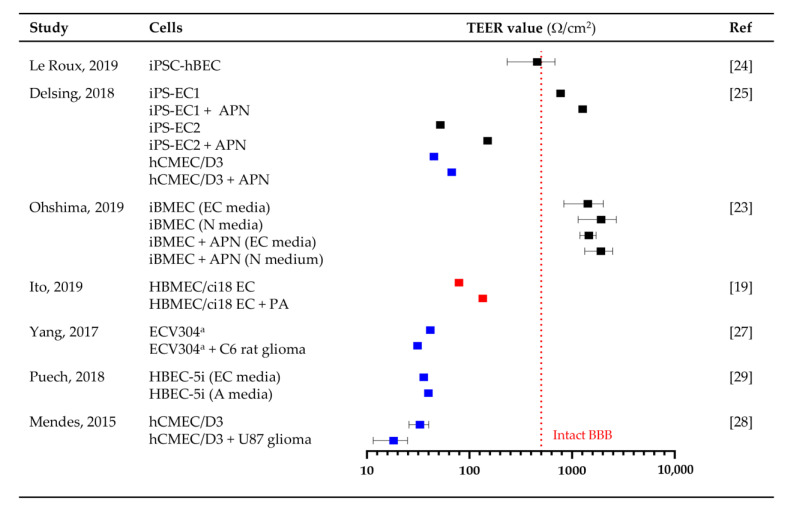
In vitro TEER values. Forest plot shows TEER values determined for transwell models using stem (iPSC; ■), primary (■) and immortalized ECs (■) with or without astrocyte (A), pericytes (P) and neurons (N). Symbols show mean ± SD for the reported experiments. ^a^ ECV304 was later identified to be a human urinary bladder carcinoma cell line, presenting many endothelial cell (EC) phenotypic characteristics [30]. A threshold TEER value of ≥500 Ω/cm^2^ (denoted by dotted red line) has been proposed to indicate an intact BBB [22]. hBMEC/hCMEC, human brain/cerebral microvascular endothelial cell; iBMEC, induced brain microvascular endothelial cell; iPSC, induced pluripotent stem cell.

TEER measurements were only reported for transwell models. Of the seven transwell models that employed TEER assessments of barrier integrity, three (42.9%) utilized 6 or 12 mm chamber electrodes, two (28.6%) utilized “chopstick” electrodes and two (28.6%) did not specify the type of electrode used. The reported TEER values include a range of monoculture, co-culture and stem, primary and immortalized ECs and are tabulated in Table S4. Four (57.1%) studies utilized immortalized EC monocultures (ECV304, HBEC-5i and hCMEC/D3) noting low TEER values of ~40 Ω/cm^2^ indicating comparatively leaky barriers. Primary ECs were used in one (14.3%) study producing TEER values of ~80 Ω/cm^2^. The highest TEER values (>400 Ω/cm^2^) were found in the three (42.9%) studies that utilized iPSC-EC cells (Figure 3). This suggests that only transwell models employing iPSC-ECs would be deemed physiologically relevant BBB models (Figure 3). However, variation will be introduced by the TEER methodology (chamber vs. “chopstick”) and day of testing used.

EC–astrocyte co-cultures and EC–pericyte–astrocyte tricultures consistently exhibited ~1.5–3-fold higher TEER values than EC monocultures (Figure 3). Indeed, BBB barrier integrity could be improved by the addition of astrocyte- or neuron-conditioned media. Using two HBEC-5i EC monoculture models, Puech and colleagues showed that human astrocyte-conditioned media achieved and stabilized BBB barrier integrity in 14 days, compared to 19 days with standard medium and had lower BBB permeability [29]. In a similar study, Ohshima and colleagues showed that neuron-conditioned media significantly increased iBMEC monoculture barrier integrity (>1000 Ω/cm^2^) compared to EC medium alone [23].

Barrier integrity is altered by several experimental conditions, including the type of transwell insert, the cell culture conditions and the order of cells in co-culture. The surface area, pore size and composition of transwell membranes can alter barrier integrity. Stone and colleagues found that the addition of pericytes to 24-well plates had poorer results and barrier integrity compared to the 12-well plates due to lower surface area [20]. They also noted that 3.0 μm pore size had higher TEER values than 0.4 μm inserts, suggesting that increased contact between cells resulted in greater barrier integrity [20]. Barrier integrity was higher in spontaneously differentiated iPSCs than VEGF-induced differentiation in both monoculture and co-culture transwell models [28]. Of note, while iPSC-ECs mimic in vivo physiology, they required a larger number of cells and time to establish; 1 × 10^6^ iPSCs give rise to 7.5 × 10^6^ ECs in 20 days following mitogen or spontaneous differentiation [17,41].

In a tri-culture model, barrier integrity was influenced by cell order and orientation, with EC–pericyte–astrocyte tri-cultures reporting the highest TEER value [19]. ECs on the apical side of a transwell insert and a mixed primary astrocyte–pericyte culture on the basolateral side exhibited significantly higher and more stable TEER values compared to astrocytes or pericytes alone [20]. The inclusion of pericytes increased the baseline TEER value in this study from 30 to 40 Ω/cm^2^ [20]. By incorporating glioma cells into the BBB, the impact of tumor cells on permeability can be assessed (termed BBTB models). In BBTB models, the inclusion of human immortalized GBM cell line U87 or C6 rat glioma cells reduced BBB TEER by 25–34%, suggesting a leakier barrier consistent with clinical pathology [28,30].

#### 3.3.3. Junctional Protein and Transporter Expression

**Figure 4:**
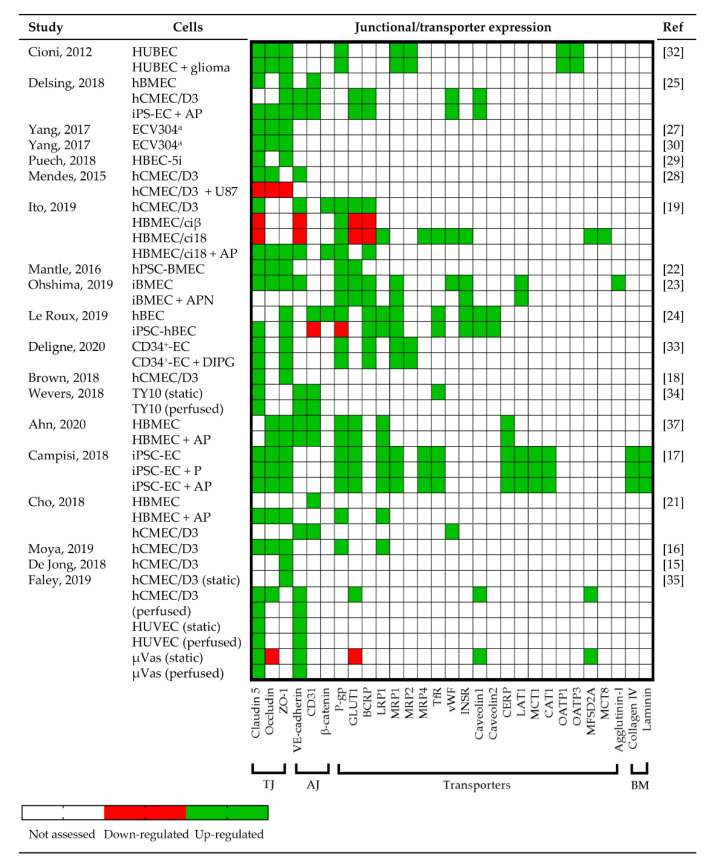
Junctional, adherens and transporter expression. A heat map of the expression of up- (green) and down- (red) regulated expression as assessed by immunofluorescent staining and/or real-time quantitative reverse transcription polymerase chain reaction. Proteins/genes that were not assessed are denoted in white. ^a^ ECV304 was later identified to be a human urinary bladder carcinoma cell line, presenting many endothelial cell (EC) phenotypic characteristics [30]. µVas, microvascular; A, astrocyte; AJ, adherens junction; BCRP, breast cancer resistance protein; BM, basement membrane; CAT1, cationic amino acid transporter 1; CERP, cholesterol efflux regulatory protein; DIPG, diffuse intrinsic pontine glioma; EC, endothelial cell; GLUT1, glucose transporter 1; hBEC/hBMEC/hCMEC/ HUBEC, human brain/cerebral (microvascular) endothelial cell; HUVEC, human umbilical vein endothelial cell; iBMEC, induced brain microvascular endothelial cell; INSR, insulin receptor; iPSC, induced pluripotent stem cell; LAT1, L-type / large neutral amino acid transporter 1; LRP1, low-density lipoprotein receptor-related protein 1; MCT, monocarboxylate transporter 1; MRP, multi-drug resistance protein; P, pericyte; P-gp, P-glycoprotein protein; OATP, organic anion transporter polypeptide 1; TfR, transferrin receptor; TJ, tight junction; VE-cadherin, vascular endothelial cadherin; vWF, von Willebrand factor; ZO-1, zonulae occludens-1.

Tight and adherens junction proteins are necessary for BBB formation, integrity and function. Nineteen (79.2%) of the in vitro BBB models validated and compared the expression of tight junctional proteins ZO-1 (17; 89.5%), claudin-5 (16; 84.2%) and occludin (12; 63.2%); adherens junctional proteins vascular endothelial (VE)-cadherin (8; 42.1%), CD31 (5; 26.3%) and β-catenin (2; 10.5%); efflux transporters P-glycoprotein (P-gp; 9, 47.4%); and specific transporters including glucose transporter 1 (GLUT1; 7, 36.8%), multi-drug resistance protein 1 (MRP1; 5, 26.3%), breast cancer resistance protein (BCRP; 5, 26.3%) and low-density lipoprotein receptor-related protein 1 (LRP1; 5, 26.3%) (Figure 4 and Table S5) using confocal microscopy of immunocytochemistry, immunohistochemistry and/or quantitative real-time polymerase chain reaction.

Ito and colleagues demonstrated that primary HBMEC express claudin-5, VE-cadherin, P-gp, GLUT1 and BCRP, but immortalization downregulated these proteins [19]. The expression of claudin5, occludin, ZO-1, β-catenin, P-gp and BCRP was restored in the immortalized line HBMEC/ci18 when co-cultured with pericytes and astrocytes [19]. In contrast, the expression of transporters in iPSC-hBECs was comparable to primary brain ECs (Figure 4) [23,24], while HUVECs were least like primary brain ECs [23]. Co-cultures of ECs, astrocytes and pericytes had significantly upregulated protein or mRNA expression of tight and adherens proteins, and transporters, irrespective of whether iPSC, primary or immortalized cells lines were used [17,19,25,37] (Figure 4). Gene expression patterns of occludin were ~5 fold higher in iPSC-derived BMECs and showed greater localization to EC clefts, compared to HUVECs and μVas [35]. Similarly, the expression of CD31 (also known as PECAM-1), VE-cadherin, von Willebrand factor and caveolin1 staining was higher in iPS-EC2, but occludin staining was more prominent in iPS-EC1 [25].

In some studies, the expression pattern of tight junction proteins was diffuse within the cytoplasm rather than expressed on the plasma membrane [19,30], consistent with the recent discovery of novel non-tight junction roles for these proteins—RNA metabolism and nuclear function [42]. When glioma cells were included in the BBTB model, there was a ~40% reduction in tight and adherens protein expression, uncontrolled cell growth, loss/destabilization of tight and adherens junctions at the interendothelial cleft and a loss of barrier integrity [28]. Destabilization of the BBB was associated with inflammatory factors, such as interleukin-6, interleukin-8 and VEGF-A, produced by the glioma cells [21,28].

Brown and colleagues demonstrated that shear stress did not significantly change the cell morphology or expression of tight junction proteins in immortalized hCMEC/D3 cells, indicating that tight junctions are preserved in flow models [18]. Increasing shear stress conditions to in vivo physiological levels (4 dyne/cm^2^) increased barrier tightness and efflux transporter protein expression [37]. Furthermore, perfusion of TY10 ECs improved barrier formation by increasing the expression of VE-cadherin, claudin-5 and CD31 compared to static conditions [34].

#### 3.3.4. Permeability

**Figure 5:**
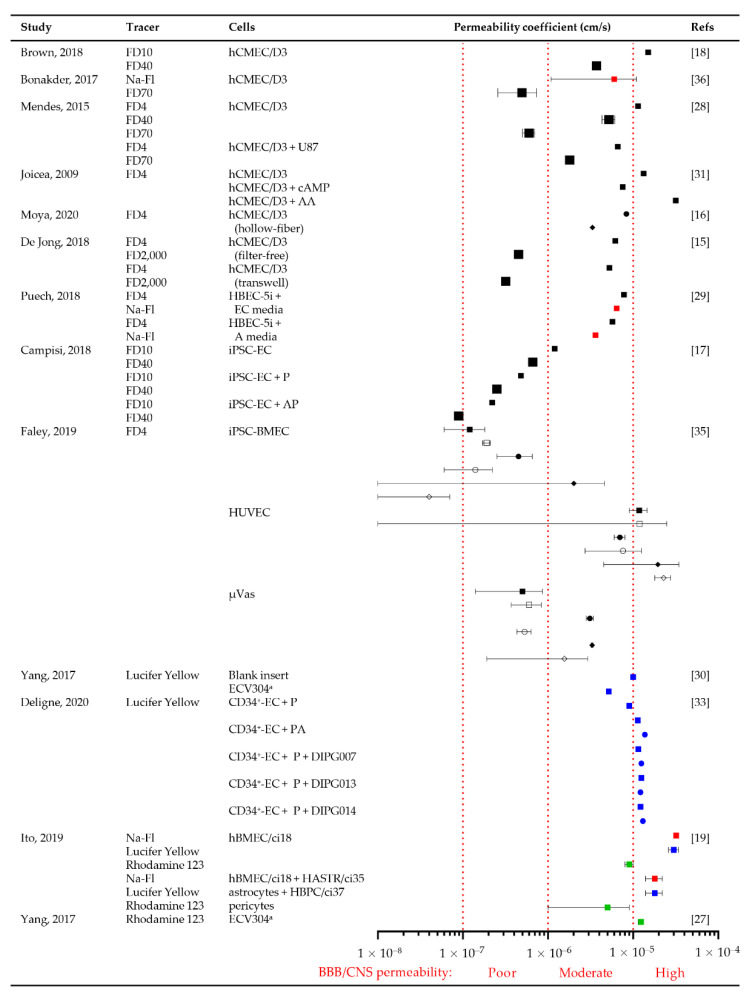
In vitro permeability coefficient values for fluorescent tracers. Forest plot shows permeability coefficients determined for FITC-dextran (FD ≤ 10 kDa ■ or ≥40 kDa; ■), sodium fluorescein (Na-Fl; 376 Da; ■); Lucifer Yellow (452 Da; ■) and Rhodamine123 (381 Da; ■; P-gp substrate) in in vitro BBB models using stem (iPSC), primary and immortalized ECs with or without astrocyte (A), pericytes (P) and neurons (N). Symbols show mean ± SD for the reported experiments at day 1 (■), 7 (●) and 14 (◆) and under static (■) or perfused conditions (●). ^a^ ECV304 was later identified to be a human urinary bladder carcinoma cell line, presenting many EC phenotypic characteristics [30]. High, moderate and poor CNS/BBB permeability is denoted by red dotted lines at >10^−5^, 10^−6^, and 10^−7^ cm/s, respectively.

Of the 24 articles reviewed, 13 studies used fluorescent tracers to determine BBB permeability. In vitro permeability coefficients are the rate at which a molecule crosses from the luminal (apical) surface of the BBB (membrane) to the abluminal (basal) as a factor of the surface area of the BBB and expressed in cm/s. BBB barrier integrity and permeability are inversely linked, with increasing barrier integrity (TEER values), barrier permeability (coefficient) decreases for the same sized compound. Nine (69.2%) investigated the diffusive permeability of fluorescently labelled FITC-dextran (4–2000 kDa), three (23.1%) lucifer yellow (245 Da), three (23.1%) sodium-fluorescein (376 Da) and two (15.4%) the active permeability of P-gp substrate Rhodamine 123 (380.8 Da) (Figure 5 and Table S6). In vivo rat cerebral microcirculation studies utilizing carotid artery perfusion reported permeability coefficients of 3.1 ± 1.3 × 10^−7^ [43], 1.37 ± 0.26 × 10^−7^ cm/s [44] and 0.15 × 10^−6^ cm/s [45] for 10 kDa, 40 kDa and 70 kDa FITC-dextran, respectively. Most fluorescent tracer studies reported permeability coefficients higher than 3.31 ± 0.24 × 10^−7^ cm/s (Figure 5), the proposed cutoff for “normal” permeability of sodium fluorescein (376 Da) in vitro [22], suggesting greater than “normal physiological” permeability.

Seven studies (63.6%) used immortalized ECs (hCMEC/D3, HBEC-5i and ECV304). Permeability was inversely correlated with tracer size (molecular weight) [18,28,29]. The lowest permeability was detected in hollow-fiber followed by filter-free and transwell models [15,16], indicating tighter barrier integrity. Compared to immortalized EC monocultures (~1.3 × 10^−5^ cm/s), stem cell-derived ECs had lower permeability coefficients (1.2 × 10^−7^ cm/s) indicating greater BBB integrity for induced ECs (Figure 5).

The inclusion of glioma cells (e.g., U87) increased BBTB permeability 2-3-fold compared to hCMEC/D3 monocultures [28] (Figure 5). Similarly, CD34^+^-ECs cultured with pericyte-glioma (DIPG-007, DIPG-013 and DIPG-014) BBTB were more permeable than EC–pericyte–astrocyte BBBs [33].

As with the TEER barrier integrity assessments, the addition of astrocyte-conditioned media reduced immortalized EC monoculture permeability [29], whilst the addition of pericytes and pericyte–astrocyte co-cultures to iPSC-ECs further decreased BBB permeability by ~2.5- and 2.2-fold, respectively [17]. iPSC-BMECs and µVas cells showed decreased permeability compared to static conditions [35] due to reduced contact time with the “vessel” wall to enable passive diffusion. Permeability was not influenced by P-gp efflux transport activity, as there was no change in activity due to the increasing perfusion rate (100, 300 or 1000 μL/min) [35]. In contrast, large vessel HUVECs showed increased permeability with perfusion.

A direct comparison of human and non-human primary EC monocultures reported that BBB permeability coefficients for primary bovine ECs are more comparable to human ECs than primary rat ECs [46]. However, the incubation of human iPSCs with bovine-derived 1% platelet poor plasma reduces human BBB barrier integrity [23].

The two induced stem cell EC studies that reported TEER values >500 Ω/cm^2^ (Figure 3) did not report permeability coefficients to enable further interrogation of these “proposed” thresholds [23,25].

**Figure 6:**
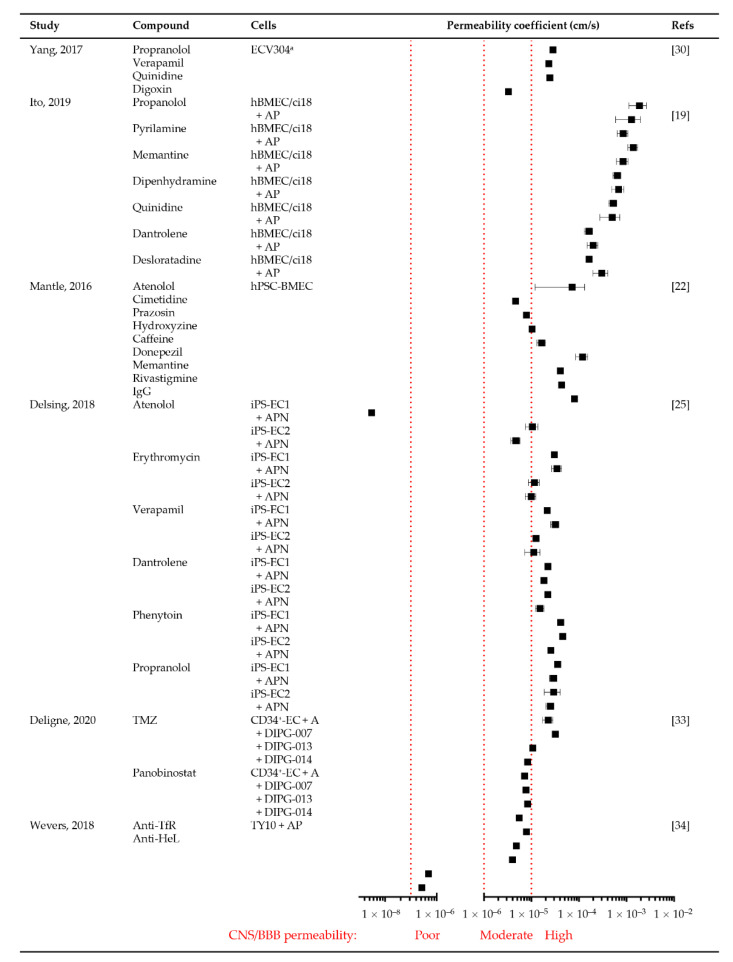
In vitro permeability coefficient values for drugs. Forest plot shows permeability coefficients determined in in vitro BBB models using stem (iPSC), primary and immortalized ECs with or without astrocyte (A), pericytes (P) and neurons (N). Compound molecular weights are presented in Table S4. Symbols show mean (■) ± SD for the reported experiments. ^a^ ECV304 was later identified to be a human urinary bladder carcinoma cell line, presenting many EC phenotypic characteristics [30]. High, moderate and poor CNS/BBB permeability is denoted by red dotted lines at >10^−5^, 10^−6^ and 10^−7^ cm/s, respectively.

A further six studies utilized compounds or drugs of known molecular weight to assess the permeability of the in vitro BBB/BBTB (Table S6). These included small hydrophilic (e.g., urea, mannitol and atenolol) and small hydrophobic compounds (e.g., inulin), as well as known CNS permeable and impermeable drugs, chemotherapies and antibodies (Figure 6).

While EC monoculture BBB models could delineate compounds and drugs of low and high CNS permeability [22,30], EC–astrocyte–pericyte tri-culture models had superior permeability profiles and lower permeability values of efflux transporter substrates [19] (Table S4). Using immortalized hBMEC/ci18 EC, HASTR/ci35 astrocyte, HBPC pericyte tri-cultures, Ito and colleagues determined a poor BBB permeability threshold of 18 × 10^−6^ cm/s [19], comparable to the CNS permeability threshold of 15 × 10^−6^ cm/s derived by Mantle and colleagues using hPSC-derived EC monocultures and CNS-permeable Alzheimer’s drugs [22] (Figure 6). Using a BBB and BBTB model, Deligne and colleagues demonstrated comparable permeability coefficients for CD34^+^-EC and astrocyte versus CD34+-EC and DIPG co-cultures, with CNS permeable TMZ more permeable than CNS impermeable panobinostat in all cultures, except for DIPG-007 [33] (Figure 6). This reflects the heterogeneity of patient tumors and derived primary cell lines.

Models of IgG antibodies (~150 kDa) were also tested, with a stable permeability of 2.99 ± 0.64 × 10^−9^ cm/s for BBBs with a TEER threshold of >900 Ω/cm^2^ [22] (Figure 6). The transferrin receptor (TfR) is a pivotal receptor on the brain microvascular endothelium for receptor-mediated transcytosis. The transcellular passage of mouse anti-human TfR antibody (MEM-189; IgG1) was significantly higher in EC–astrocyte–pericyte tri-cultures compared to EC monocultures [34] (Figure 6). Similarly, permeability data utilizing the in vitro spheroidal tri-culture BBB model recapitulated the extravasation of Angiopep-2, the ligand for the LRP1 transporter, through the BBB and into the brain parenchyma of mice following intravenous tail injection [21]. These studies demonstrate a critical role for the inclusion of astrocytes/pericytes in BBB models when assessing the permeability of drugs/compounds requiring receptor-mediated transcytosis.

Mantle and colleagues reported a concordance of permeability for seven drugs measured using an in vitro hPSC-BMECs BBB and those obtained from mice in vivo, noting that the in vitro BBB was more restrictive (lower permeability) than in vivo measures [22]. Using in vitro and in vivo BBB models of neonatal (2 weeks old) and adult (8 weeks old) rats, Takata and colleagues demonstrated that in vitro BBB values are comparable to the in situ/in vivo trans cardiac brain perfusion neonatal and adult blood-to-brain influx values for nicotine and inulin, but not valproic acid due to a more efficient efflux than influx transport pathway [47]. However, it was noted that brain influx permeabilities decreased with animal age, indicating that the age of small animals used in in vitro and in vivo BBB permeability research is important.

### 3.4. Comparison of In Vitro BBB/BBTB Models with Clinical Data

Critical assessment of the ability of in vitro and in vivo models to predict patient BBB/CNS permeability in the preclinical phase will improve the translation of novel therapeutics for high-grade glioma into a clinical setting. Ohshima and colleagues used a transwell model to assess the ability of iPSC-BMEC and rat primary BMECs to predict human patient CSF/plasma permeability [23]. They found that iPSC-BMEC monoculture and EC–astrocyte–pericyte–neuron co-culture models showed a strong correlation with low CNS permeability (monoculture, *R*^2^ = 0.49; co-culture, *R*^2^ = 0.60), but rat primary BMECs had a stronger correlation for all seven drugs assessed (*R*^2^ = 0.73) [23]. Similarly, Le Roux and colleagues found that the drug permeability across the in vitro iPSC-ECs BBB transwell model and rate of patient plasma to brain permeability via positron emission (K_1_) were highly correlated (*R*^2^ = 0.83) for drugs of low, moderate and high CNS permeability [24].

Generally, stem cell-derived and primary cells were noted to have more replicative human in situ BBB permeability. This has precedence in the in vivo setting. The immortalized cell line U87 had 2.5- to 4-fold higher contrast-enhancer uptake detected by magnetic resonance imaging than a patient-derived cell line or 16 glioma patient tumors [48]. This indicates substantially greater BBB disruption in the immortalized xenograft.

## 4. Discussion

This systematic review aimed to support academic and pharmaceutical researchers in the design of in vitro BBB/BBTB platforms to provide sufficient in vivo and clinically relevant data to have confidence that the drugs are brain permeable and, therefore, are suitable for future therapeutic efficacy studies. By accurately quantifying drug permeability to the brain, this may enable modifications to be made to drugs at an early stage in drug development to improve their translation into patients, such that better tumor control and patient survival can be achieved.

In performing this systematic review, we asked whether in vitro human BBB and tumor (BBTB) are suitable alternatives to “gold standard” in vivo models to determine the permeability of novel therapeutics to treat brain cancer. The search examined 24 articles from the last 5 years regarding which in vitro model was used, the cell origin (stem cell-derived, primary or immortalized), monoculture vs. co-culture and other parameters that affected model success.

Tri-culture microfluidic in vitro models proved to be the most replicative of the physiological environment reported in vivo. It is also able to successfully replicate shear stress conditions without compromising barrier integrity and allows the precise and flexible control of the cellular environment. iPSC-EC models have been noted to exhibit morphological and physiological characteristics closest to in vivo characteristics, with comparable barrier integrity, protein and transporter expression and barrier permeability. It should be highlighted that pluripotent-derived endothelial cells should be confirmed as bona fide endothelial cells before use in in vitro model systems [49]. The inclusion of multiple cell types, including ECs, astrocytes, pericytes and neurons, improved barrier integrity, and the conditioning of these cells in brain-specific media (i.e., astrocyte and neuron) further strengthened the barrier. The inclusion of primary GBM or DIPG glioma cells was more comparable to human BBTBs, compared to immortalized cell lines. Important minimum thresholds to achieve include a TEER value of >500 Ω/cm^2^ for an intact BBB, permeability value of >15–18 × 10^−6^ cm/s and shear stress of 3–4 dyne/cm^2^. A review of current microfluidic models used for BBB research is available from Jiang and colleagues [50].

A number of practical limitations exist for this ideal in vitro model. Access to microfluidic devices is not available in all laboratories, as they need to be purchased or 3D printed (where possible) and are arguably more technically complex to assemble compared to the more common models using transwell co-culture systems. The use of primary/stem cells also requires both access to fresh tissue (e.g., close proximity to hospitals) and human ethics approval.

Of note, our systematic review asked whether human in vitro BBB models can mimic in vivo (largely rodent) data. However, interspecies differences between human and rodent ECs, and therefore, BBB/BBTB, in vitro and in vivo will invariably exist. In vivo models commonly employ human primary or immortalized cell xenografts into murine microenvironments for the assessment of in vivo permeability, which may cause the under- or overestimation of drug permeability and therapeutic efficacy due to interspecies compatibility hindering clinical translation. Furthermore, transcriptomic data from the laser capture microdissection of normal human and mouse brain ECs and pericytes revealed the enrichment of human (211) and murine (142) microvessel genes [51]. Critically, there was differential expression of solute carrier (SLC) and ATP-binding cassette (ABC) genes, including those encoding P-gp and BCRP. Given the role of these transporters in drug delivery and efflux transport, interspecies differences remain an important consideration in BBB/BBTB permeability assessments, and more research in this area is needed.

## 5. Conclusions

This systematic review revealed that in vitro models are a suitable replacement for current in vivo models in their ability to replicate barrier integrity, protein and transporter expression and permeability akin to human BBB/BBTBs. The models that most closely mimic in vivo drug penetrance incorporated multi-cell co-cultures in microfluidic systems. However, the increased complexity that these introduce may not be suitable for all studies. Thus, understanding the merits of the different model systems employed will aid the researcher in planning drug permeability assessments and ultimately lead to better in vitro to in vivo correlations.

## Figures and Tables

**Table 1:** Advantages and disadvantages of in vitro BBB/BBTB models.

Model	Advantages	Disadvantages	Refs
Transwell	Reproducible Scalability BBB functionality and practicability Easy, simple cell culture setup Easy to control Allows access to both apical and basal compartments for therapeutic testing Allows visualization of cells for the duration of the experimental timeline Uses minimal resources Versatile	Limited mimicking of BBB and microenvironmental features, e.g., cell–cell/cell–matrix interactions Lack of accurate brain capillary models due to inefficient junctional protein and membrane transporter expression Modification of culture conditions necessary for each model Improvement of barrier tightness and efflux functionality necessary ECs cannot form tight junctions along inner apical chamber wall which causes incomplete coverage of transwell inserts at monolayer perimeter (“edge effect”) No 3D cellular organization No direct cell–cell contact ECs can distribute unevenly on inserts, causing imperfect barriers Requires large number of cells	[17,18,19,20,21,22,23,24,25,26,27,28,29,30,31,32,33]
Microfluidic	Precise control of cellular and extracellular environment Mimic structures and interactions found in vivo More physiologically relevant morphology Different cell types can easily be incorporated into device Can include additional features, e.g., growth factors, differentiation factors, etc. Cell type ratios can be modified to explore different regions of the brain Can be modified to explore healthy and diseased brain states Can assess physiological flow and shear stress conditions	Current models have larger vessel diameters (~100–800 μm) than in vivo BBB vasculature (capillaries ~7–10 μm) Do not realistically recreate in vivo BBB microvasculature morphology and function, which alters transport exchange mechanisms Permeability measurements limited to quantifying fluorescent tracer concentrations Non-specific protein and small hydrophobic molecule adsorption during long-term interaction Complex assembly Expensive Requires commercial purchase or 3D printing capabilities	[17,18,20,21,34,35,36,37]
Spheroid	More accurate representation of 3-dimensional in vivo environment, than 2-dimensional cultures Cost effective Cells types in direct contact Requires lower number of cells Reproducible High-throughput, scalable Few reagents necessary to establish model	Limited ability to mimic BBB morphology and physiology Difficult to assemble Can be expensive to establish compared to transwell Cannot simulate physiological flow and shear stress	[17,20,21]
Hollow-fiber	Can mimic shear stress and physiological flow conditions Long term cell culture Easy to recover cell samples Versatile Easy to reconfigure Same platform device can be used for experiments of varying complexity Cylindrical—no sidewalls, no leaky edges Allows non-invasive observation Fiber thickness more closely mimics in vivo thickness of vessel walls	Can be difficult to extract cell samples in some device designs More commonly support 2D cell cultures	[16]
Filter-free	Better cell–cell interactions without filter membrane causing separation More physiologically relevant	Limited working distance of high magnification microscopy limits image acquisition due to >2 mm thickness of collagen gel and use of conventional well plate	[15]
Hydrogel scaffold	Hydrogels mimic many aspects of the natural extracellular matrix Can observe cell behavior in a more physiology-mimicking, 3D environment	Channel sizes are still larger than in vivo vessel diameters	[35]

BBB, blood–brain barrier.

**Table 2:** In vivo BBB/BBTB models and their advantages and disadvantages.

Model	Advantages	Disadvantages	Refs
Single/Internal Carotid Artery Perfusion	Increased accuracy in observing physiological responses Control of perfusate composition, rate and total time Reduced peripheral metabolism	Reduce clearance Limit metabolic events Long perfusion time (~60 s) may alter BBB integrity Requires brain harvest and secondary analyses	[18,25]
Intravenous Tail or Cannulated Femoral Vein Injections	Simple and efficient technique Increased accuracy of drug diffusion and clearance in physiological conditions	Rapid metabolism of therapeutic agents causes clearance before reaching brain microcirculation Metabolism-induced artefacts Requires brain harvest and secondary analyses	[18,25]
Intracerebral Microdialysis or Cerebral Open Flow Microperfusion	Technically difficult—microdialysis probe inserted into the brain Measures drug concentration in CSF or brain extracellular fluid	Requires absorption through microdialysis probe Requires liquid chromatography or mass spectrophotometry of dialysate	[38]
Primary Cell-Derived Xenograft	Increased accuracy in observing physiological responses Human tumor cells planted into an animal microenvironment may alter barrier integrity	Performed on immunodeficient mice, so immune system not considered in pathogenesis	[25,33]
Genetically Engineered Mouse Model	Genetic alterations can be specific and clearly identifiable Can replicate complete tumor development with correct location and brain kinetics Accurate information about tumor vascular networks can be gained	Host adaptations during tumor development can cause misidentification of molecular components involved in tumor pathogenesis Long tumor development period	[33]

BBB, blood–brain barrier; CSF, cerebrospinal fluid.

**Table 3:** Vessel diameters of 3D microfluidic BBB model.

Culture Type	Lateral Vessel Diameter (μm)	Transverse Vessel Diameter (μm)	Vascular Branch Length (μm)
EC	108 ± 14	29 ± 10	226 ± 40
EC + A	64 ± 13	27 ± 7	179 ± 31
EC + AP	42 ± 13	25 ± 6	136 ± 24

Data are reported as mean *±* SD for *N* = 30 microvessels. A, astrocyte; EC, endothelial cell. P, pericyte. Data obtained from [17].

## Data Availability

No new data were created or analyzed in this study. Data sharing is not applicable to this article.

## Supplementary Materials

The following are available online at https://www.mdpi.com/2072-6694/13/5/955/s1, Table S1: PRISMA Checklist, Table S2: Number of articles identified from keyword search results, Table S3: Cell lines, advantages and disadvantages of in vitro BBB/BBTB model systems, Table S4: TEER values for transwell in vitro BBB/BBTB models, Table S5: Junctional protein and efflux transporter expression in in vitro BBB/BBTB models, Table S6: Permeability coefficients for in vitro BBB/BBTB models.

## References

[1] Suryadevara R., Fadel H., Michelhaugh S.K., Mittal S., Parajuli P., Kesharwani P., Gupta U. (2018). Tumors of the Central Nervous System: Anatomy and Interventional Considerations. Nanotechnology-Based Targeted Drug Delivery Systems for Brain Tumors.

[2] Bhowmik A., Khan R., Ghosh M.K. (2015). Blood Brain Barrier: A Challenge for Effectual Therapy of Brain Tumors. BioMed Res. Int..

[3] Erhart F., Hackl M., Hahne H., Buchroithner J., Meng C., Klingenbrunner S., Reitermaier R., Fischhuber K., Skalicky S., Berger W. (2020). Combined proteomics/miRNomics of dendritic cell immunotherapy-treated glioblastoma patients as a screening for survival-associated factors. npj Vaccines.

[4] Noureldine M.H.A., Shimony N., Jallo G.I., Jallo G.I., Noureldine M.H.A., Shimony N. (2020). Diffuse Midline Glioma—Diffuse Intrinsic Pontine Glioma. Brainstem Tumors.

[5] Tivnan A., Heilinger T., Lavelle E.C., Prehn J.H.M. (2017). Advances in immunotherapy for the treatment of glioblastoma. J. Neuro-Oncology.

[6] Haddad-Tóvolli R., Dragano N.R.V., Ramalho A.F.S., Velloso L.A. (2017). Development and Function of the Blood-Brain Barrier in the Context of Metabolic Control. Front. Neurosci..

[7] Abbott N.J., Rönnbäck L., Hansson E. (2006). Astrocyte–endothelial interactions at the blood–brain barrier. Nat. Rev. Neurosci..

[8] Hawkins B.T., Davis T.P. (2005). The Blood-Brain Barrier/Neurovascular Unit in Health and Disease. Pharmacol. Rev..

[9] Komarova Y.A., Kruse K., Mehta D., Malik A.B. (2017). Protein Interactions at Endothelial Junctions and Signaling Mechanisms Regulating Endothelial Permeability. Circ. Res..

[10] Arvanitis C.D., Ferraro G.B., Jain R.K. (2020). The blood–brain barrier and blood–tumour barrier in brain tumours and metastases. Nat. Rev. Cancer.

[11] Muoio V., Persson P.B., Sendeski M.M. (2014). The neurovascular unit - concept review. Acta Physiol..

[12] Van Tellingen O., Yetkin-Arik B., de Gooijer M., Wesseling P., Wurdinger T., de Vries H. (2015). Overcoming the blood–brain tumor barrier for effective glioblastoma treatment. Drug Resist. Updat..

[13] Passeleu-Le Bourdonnec C., Carrupt P.-A., Scherrmann J.M., Martel S. (2013). Methodologies to Assess Drug Permeation Through the Blood–Brain Barrier for Pharmaceutical Research. Pharm. Res..

[14] Moher D., Liberati A., Tetzlaff J., Altman D.G. (2009). Preferred Reporting Items for Systematic Reviews and Meta-Analyses: The PRISMA Statement. PLoS Med..

[15] De Jong E., Williams D.S., Abdelmohsen L.K.E.A., Van Hest J.C.M., Zuhorn I.S. (2018). A filter-free blood-brain barrier model to quantitatively study transendothelial delivery of nanoparticles by fluorescence spectroscopy. J. Control. Release.

[16] Moya M.L., Triplett M., Simon M., Alvarado J., Booth R., Osburn J., Soscia D., Qian F., Fischer N.O., Kulp K. (2019). A Reconfigurable In Vitro Model for Studying the Blood–Brain Barrier. Ann. Biomed. Eng..

[17] Campisi M., Shin Y., Osaki T., Hajal C., Chiono V., Kamm R.D. (2018). 3D self-organized microvascular model of the human blood-brain barrier with endothelial cells, pericytes and astrocytes. Biomaterials.

[18] Brown T.D., Nowak M., Bayles A.V., Prabhakarpandian B., Karande P., Lahann J., Helgeson M.E., Mitragotri S. (2018). A microfluidic model of human brain (μHuB) for assessment of blood brain barrier. Bioeng. Transl. Med..

[19] Ito R., Umehara K., Suzuki S., Kitamura K., Nunoya K.-I., Yamaura Y., Imawaka H., Izumi S., Wakayama N., Komori T. (2019). A Human Immortalized Cell-Based Blood–Brain Barrier Triculture Model: Development and Characterization as a Promising Tool for Drug−Brain Permeability Studies. Mol. Pharm..

[20] Stone N.L., England T.J., O’Sullivan S.E. (2019). A Novel Transwell Blood Brain Barrier Model Using Primary Human Cells. Front. Cell. Neurosci..

[21] Cho C.-F., Wolfe J.M., Fadzen C.M., Calligaris D., Hornburg K., Chiocca E.A., Agar N.Y.R., Pentelute B.L., Lawler S.E. (2017). Blood-brain-barrier spheroids as an in vitro screening platform for brain-penetrating agents. Nat. Commun..

[22] Mantle J.L., Min L., Lee K.H. (2016). Minimum Transendothelial Electrical Resistance Thresholds for the Study of Small and Large Molecule Drug Transport in a Human in Vitro Blood–Brain Barrier Model. Mol. Pharm..

[23] Ohshima M., Kamei S., Fushimi H., Mima S., Yamada T., Yamamoto T. (2019). Prediction of Drug Permeability Using In Vitro Blood–Brain Barrier Models with Human Induced Pluripotent Stem Cell-Derived Brain Microvascular Endothelial Cells. BioResearch Open Access.

[24] Le Roux G., Jarray R., Guyot A.-C., Pavoni S., Costa N., Théodoro F., Nassor F., Pruvost A., Tournier N., Kiyan Y. (2019). Proof-of-Concept Study of Drug Brain Permeability Between in Vivo Human Brain and an in Vitro iPSCs-Human Blood-Brain Barrier Model. Sci. Rep..

[25] Delsing L., Dönnes P., Sánchez J., Clausen M., Voulgaris D., Falk A., Herland A., Brolén G., Zetterberg H., Hicks R. (2018). Barrier Properties and Transcriptome Expression in Human iPSC-Derived Models of the Blood-Brain Barrier. STEM CELLS.

[26] Kulczar C., Lubin K.E., Lefebvre S., Miller D.W., Knipp G.T. (2017). Development of a direct contact astrocyte-human cerebral microvessel endothelial cells blood–brain barrier coculture model. J. Pharm. Pharmacol..

[27] Yang S., Mei S., Jin H., Zhu B., Tian Y., Huo J., Cui X., Guo A., Zhao Z. (2017). Identification of two immortalized cell lines, ECV304 and bEnd3, for in vitro permeability studies of blood-brain barrier. PLoS ONE.

[28] Mendes B., Marques C., Carvalho I., Costa P., Martins S., Ferreira D., Sarmento B. (2015). Influence of glioma cells on a new co-culture in vitro blood–brain barrier model for characterization and validation of permeability. Int. J. Pharm..

[29] Puech C., Hodin S., Forest V., He Z., Mismetti P., Delavenne X., Perek N. (2018). Assessment of HBEC-5i endothelial cell line cultivated in astrocyte conditioned medium as a human blood-brain barrier model for ABC drug transport studies. Int. J. Pharm..

[30] Yang S., Jin H., Zhao Z. (2017). An ECV304 monoculture model for permeability assessment of blood–brain barrier. Neurol. Res..

[31] Joice S.L., Mydeen F., Couraud P.-O., Weksler B.B., Romero I.A., Fraser P.A., Easton A.S. (2009). Modulation of blood–brain barrier permeability by neutrophils: In vitro and in vivo studies. Brain Res..

[32] Cioni C., Turlizzi E., Zanelli U., Oliveri G., Annunziata P. (2012). Expression of Tight Junction and Drug Efflux Transporter Proteins in an in vitro Model of Human Blood–Brain Barrier. Front. Psychiatry.

[33] Deligne C., Hachani J., Duban-Deweer S., Meignan S., Leblond P., Carcaboso A.M., Sano Y., Shimizu F., Kanda T., Gosselet F. (2020). Development of a human in vitro blood–brain tumor barrier model of diffuse intrinsic pontine glioma to better understand the chemoresistance. Fluids Barriers CNS.

[34] Wevers N.R., Kasi D.G., Gray T., Wilschut K.J., Smith B., Van Vught R., Shimizu F., Sano Y., Kanda T., Marsh G. (2018). A perfused human blood–brain barrier on-a-chip for high-throughput assessment of barrier function and antibody transport. Fluids Barriers CNS.

[35] Faley S.L., Neal E.H., Wang J.X., Bosworth A.M., Weber C.M., Balotin K.M., Lippmann E.S., Bellan L.M. (2019). iPSC-Derived Brain Endothelium Exhibits Stable, Long-Term Barrier Function in Perfused Hydrogel Scaffolds. Stem Cell Rep..

[36] Bonakdar M., Graybill P.M., Davalos R.V. (2017). A microfluidic model of the blood–brain barrier to study permeabilization by pulsed electric fields. RSC Adv..

[37] Ahn S.I., Sei Y.J., Park H.-J., Kim J., Ryu Y., Choi J.J., Sung H.-J., Macdonald T.J., Levey A.I., Kim Y. (2020). Microengineered human blood–brain barrier platform for understanding nanoparticle transport mechanisms. Nat. Commun..

[38] Sharma B., Luhach K., Kulkarni G.T., Gao H., Gao X. (2019). 4—In vitro and in vivo models of BBB to evaluate brain targeting drug delivery. Brain Targeted Drug Delivery System.

[39] Elbakary B., Badhan R.K.S. (2020). A dynamic perfusion based blood-brain barrier model for cytotoxicity testing and drug permeation. Sci. Rep..

[40] Wong A.D., Ye M., Levy A.F., Rothstein J.D., Bergles D.E., Searson P.C. (2013). The blood-brain barrier: An engineering perspective. Front. Neuroeng..

[41] Rosa S., Praça C., Pitrez P.R., Gouveia P.J., Aranguren X.L., Ricotti L., Ferreira L.S. (2019). Functional characterization of iPSC-derived arterial- and venous-like endothelial cells. Sci. Rep..

[42] Morgan S.V., Garwood C.J., Jennings L., Simpson J.E., Castelli L.M., Heath P.R., Mihaylov S.R., Vaquéz-Villaseñor I., Minshull T.C., Ince P.G. (2018). Proteomic and cellular localisation studies suggest non-tight junction cytoplasmic and nuclear roles for occludin in astrocytes. Eur. J. Neurosci..

[43] Shi L., Zeng M., Sun Y., Fu B.M. (2014). Quantification of Blood-Brain Barrier Solute Permeability and Brain Transport by Multiphoton Microscopy. J. Biomech. Eng..

[44] Wang Y.I., Abaci H.E., Shuler M.L. (2017). Microfluidic blood-brain barrier model provides in vivo-like barrier properties for drug permeability screening. Biotechnol. Bioeng..

[45] Yuan W., Lv Y., Zeng M., Fu B.M. (2009). Non-invasive measurement of solute permeability in cerebral microvessels of the rat. Microvasc. Res..

[46] Weksler B.B., Subileau E.A., Perrière N., Charneau P., Holloway K., Leveque M., Tricoire-Leignel H., Nicotra A., Bourdoulous S., Turowski P. (2005). Blood-brain barrier-specific properties of a human adult brain endothelial cell line. FASEB J..

[47] Takata F., Dohgu S., Yamauchi A., Matsumoto J., Machida T., Fujishita K., Shibata K., Shinozaki Y., Sato K., Kataoka Y. (2013). In Vitro Blood-Brain Barrier Models Using Brain Capillary Endothelial Cells Isolated from Neonatal and Adult Rats Retain Age-Related Barrier Properties. PLoS ONE.

[48] Brighi C., Reid L., Genovesi L.A., Kojic M., Millar A., Bruce Z., White A.L., Day B.W., Rose S., Whittaker A.K. (2020). Comparative study of preclinical mouse models of high-grade glioma for nanomedicine research: The importance of producing blood-brain barrier heterogeneity. Theranostics.

[49] Lu T.M., Houghton S., Magdeldin T., Durán J.G.B., Minotti A.P., Snead A., Sproul A., Nguyen D.-H.T., Xiang J., Fine H.A. (2021). Pluripotent stem cell-derived epithelium misidentified as brain microvascular endothelium requires ETS factors to acquire vascular fate. Proc. Natl. Acad. Sci..

[50] Jiang L., Li S., Zheng J., Li Y., Huang H. (2019). Recent Progress in Microfluidic Models of the Blood-Brain Barrier. Micromachines.

[51] Song H.W., Foreman K.L., Gastfriend B.D., Kuo J.S., Palecek S.P., Shusta E.V. (2020). Transcriptomic comparison of human and mouse brain microvessels. Sci. Rep..

