# Supplementary material for: Are In Vitro Human Blood–Brain–Tumor-Barriers Suitable Replacements for In Vivo Models of Brain Permeability for Novel Therapeutics?
Source: Cancers (Basel). 2021 Feb 25;13(5):955. doi: 10.3390/cancers13050955 (PMC7956470; doi:10.3390/cancers13050955)
Supplement: Supplementary file 1 [file cancers-13-00955-s001.pdf]

Supplementary Materials

Are In Vitro Human Blood–Brain–Tumor-Barriers Suitable Replacements for In Vivo Models of Brain Permeability for Novel Therapeutics?

Archana Prashanth, Heather Donaghy, Shihani P. Stoner, Amanda L. Hudson, Helen R. Wheeler, Connie I. Diakos, Viive M. Howell, Georges E. Grau and Kelly J. McKelvey

Table S1. PRISMA Checklist**.**

Section/Topic		Checklist Item	Reported on Page #
**TITLE**			
Title	1	Identify the report as a systematic review, meta-analysis, or both.	1
**ABSTRACT**			
Structured summary	2	Provide a structured summary including, as applicable: background; objectives; data sources; study eligibility criteria, participants, and interventions; study appraisal and synthesis methods; results; limitations; conclusions and implications of key findings; systematic review registration number.	1
**INTRODUCTION**			
Rationale	3	Describe the rationale for the review in the context of what is already known.	2
Objectives	4	Provide an explicit statement of questions being addressed with reference to participants, interventions, comparisons, outcomes, and study design (PICOS).	2
**METHODS**			
Protocol and registration	5	Indicate if a review protocol exists, if and where it can be accessed (e.g., Web address), and, if available, provide registration information including registration number.	n.a.
Eligibility criteria	6	Specify study characteristics (e.g., PICOS, length of follow-up) and report characteristics (e.g., years considered, language, publication status) used as criteria for eligibility, giving rationale.	Figure 1, pg 2–3
Information sources	7	Describe all information sources (e.g., databases with dates of coverage, contact with study authors to identify additional studies) in the search and date last searched.	Figure 1, pg 2–3
Search	8	Present full electronic search strategy for at least one database, including any limits used, such that it could be repeated.	Table S1
Study selection	9	State the process for selecting studies (i.e., screening, eligibility, included in systematic review, and, if applicable, included in the meta-analysis).	Figure 1
Data collection process	10	Describe method of data extraction from reports (e.g., piloted forms, independently, in duplicate) and any processes for obtaining and confirming data from investigators.	Figure 1, pg 2–3
Data items	11	List and define all variables for which data were sought (e.g., PICOS, funding sources) and any assumptions and simplifications made.	2–3
Risk of bias in individual studies	12	Describe methods used for assessing risk of bias of individual studies (including specification of whether this was done at the study or outcome level), and how this information is to be used in any data synthesis.	3
Summary measures	13	State the principal summary measures (e.g., risk ratio, difference in means).	Figure 3–6 legends
Synthesis of results	14	Describe the methods of handling data and combining results of studies, if done, including measures of consistency (e.g., I^2) for each meta-analysis.	n.a.
Risk of bias across studies	15	Specify any assessment of risk of bias that may affect the cumulative evidence (e.g., publication bias, selective reporting within studies).	3
Additional analyses	16	Describe methods of additional analyses (e.g., sensitivity or subgroup analyses, meta-regression), if done, indicating which were pre-specified.	n.a.

**RESULTS**			
Study selection	17	Give numbers of studies screened, assessed for eligibility, and included in the review, with reasons for exclusions at each stage, ideally with a flow diagram.	Figure 1
Study characteristics	18	For each study, present characteristics for which data were extracted (e.g., study size, PICOS, follow-up period) and provide the citations.	n.a.
Risk of bias within studies	19	Present data on risk of bias of each study and, if available, any outcome level assessment (see item 12).	n.a.
Results of individual studies	20	For all outcomes considered (benefits or harms), present, for each study: (a) simple summary data for each intervention group (b) effect estimates and confidence intervals, ideally with a forest plot.	Figures 3–6
Synthesis of results	21	Present results of each meta-analysis done, including confidence intervals and measures of consistency.	n.a.
Risk of bias across studies	22	Present results of any assessment of risk of bias across studies (see Item 15).	n.a.
Additional analysis	23	Give results of additional analyses, if done (e.g., sensitivity or subgroup analyses, meta-regression [see Item 16]).	n.a.
**DISCUSSION**			
Summary of evidence	24	Summarize the main findings including the strength of evidence for each main outcome; consider their relevance to key groups (e.g., healthcare providers, users, and policy makers).	18
Limitations	25	Discuss limitations at study and outcome level (e.g., risk of bias), and at review-level (e.g., incomplete retrieval of identified research, reporting bias).	3,18
Conclusions	26	Provide a general interpretation of the results in the context of other evidence, and implications for future research.	18
**FUNDING**			
Funding	27	Describe sources of funding for the systematic review and other support (e.g., supply of data); role of funders for the systematic review.	19

Table S2. Number of articles identified from keyword search results.

Keywords	PubMed	Medline	Embase	Scopus	Filters Applied
“in vitro mode*” AND “blood brain barrier” AND “permeability”	153	175	36	201	Full text, English, Published <5 years ago
“in vivo mode*” AND “blood brain barrier” AND “permeability”	67	69	57	91	Full text, English
“in vitro mode*” AND “blood brain barrier” AND “brain cancer”	3	3	10	7	Full text, English
“in vivo mode*” AND “blood brain barrier” AND “brain cancer”	2	2	16	6	Full text, English
“in vitro mode*” AND “blood brain barrier” AND “glioblastoma”	22	21	36	30	Full text, English
“in vivo mode*” AND “blood brain barrier” AND “glioblastoma”	13	13	36	18	Full text, English
“in vitro mode*” AND “blood brain barrier” AND “glioma”	37	37	56	44	Full text, English
“in vivo mode*” AND “blood brain barrier” AND “glioma”	13	12	28	14	Full text, English
"in vitro mode*" AND "blood brain tumor barrier" AND "permeability"	1	0	1	1	Full text, English
"in vivo mode*" AND "blood brain tumor barrier" AND "permeability"	1	1	1	1	Full text, English
TOTAL	312	333	277	413	
	1335				

Searches were conducted using multiple keyword combinations in four search databases.

Table S3. Cell lines, advantages and disadvantages of in vitro BBB/BBTB model systems.

Model	Cell Types	Advantages	Disadvantages	Refs
Transwell<br>(10/19 studies)	*EC:*<br>Human EC [a]<br>ECV304 [b,d]<br>HBEC-5i [b]<br>hCMEC/D3 [b]<br>hTERT [b]<br>HBMEC/ci18 [a]<br>HBMEC [a]<br>hPSC-BMEC [c]<br>iPSC-BMEC [c]<br>iPSC-hBEC [c]<br>iPSC-EC [c]<br>CD34+-EC [c]<br>HUBEC [a]<br>*Astrocytes:*<br>HASTR/ci35 [b]<br>HBPC/ci37 [b]<br>Primary<br>iPSC-astrocytes [c]<br>*Pericytes:*<br>Primary<br>HBVP [a]<br>iPSC-pericytes [c]<br>*Neurons:*<br>Primary<br>iPSC-neurons [c]<br>*Glioma:*<br>U87<br>DIPG-007 [a]<br>DIPG-013 [a]<br>DIPG-014 [a]	• Reproducible<br>• Easy to use<br>• Scalability<br>• No extensive time- and<br>• cost-consuming labor<br>• BBB functionality and practicability<br>• Easy, simple cell culture setup<br>• Easy to control<br>• Allow access to both apical and basal compartments for therapeutic testing<br>• Allows visualization of cells for the duration of the experimental timeline<br>• Uses minimal resources<br>• Versatile	• Limited mimicking of BBB and micro-environmental features e.g., cell-cell/cell-matrix interactions<br>• Lack of accurate brain capillary models due to inefficient junctional protein and membrane transporter expression<br>• Modification of culture conditions necessary for each model<br>• Improvement of barrier tightness and efflux functionality necessary<br>• EC cannot form tight junctions along inner apical chamber wall which causes incomplete coverage of transwell inserts at monolayer perimeter<br>• Transwell inserts can be subject to "edge effects"-artificial paracellular diffusion at the perimeter of the monolayer membrane that causes leakage into side channels<br>• No 3D cellular organization<br>• No direct cell-cell contact<br>• ECs can distribute unevenly on inserts, causing imperfect barriers<br>• Requires large number of cells	[17–33]
Microfluidic<br>(5/19 studies)	*ECs:*<br>iPSC-EC [c]<br>hCMEC/D3 [b]<br>TY10 [a]<br>HBMEC [b]<br>*Astrocytes:*<br>hAst [b]<br>HA [a]<br>*Pericytes:*<br>hBPCT [b]<br>HBVP [b]	• Precise control of cellular and extracellular environment<br>• Mimic structures and interactions found in vivo<br>• More physiologically relevant morphology<br>• Different cell types can easily be incorporated into device<br>• Can include additional features e.g., growth factors, differentiation factors etc.<br>• Cell type ratios can be modified to explore different regions of the brain<br>• Can be modified to explore healthy and diseased brain states<br>• Can mimic physiological flow and shear stress conditions<br>• Supports perfusion in cell cultures	• Current models have larger vessel diameters (~100–800 μm) than in vivo BBB vasculature (capillaries ~7–10 μm)<br>• Do not realistically recreate in vivo BBB micro-vasculature morphology and function, which alters transport exchange mechanisms<br>• Permeability measurements limited to quantifying fluorescent tracer concentrations<br>• Non-specific protein and small hydrophobic molecule adsorption during long-term interaction<br>• Complex assembly<br>• Expensive<br>• Inaccessible to many laboratories	[17,18,20,21,34–37]
Spheroidal<br>(1/19 studies)	*EC:*<br>HBMEC [a]<br>hCMEC/D3 [b]<br>*Astrocytes:*<br>Primary	• More accurate representation of in vivo environment<br>• Cost effective<br>• Each cell type can interact directly with each other	• Limited ability to mimic BBB morphology and physiology<br>• Difficult to assemble<br>• Expensive compared to transwell	[17]

	Pericytes:<br>Primary	• Greater expression of BBB modulators compared to transwell<br>• Requires lower number of cells<br>• Reproducible<br>• High throughput<br>• Scalability<br>• Few reagents necessary to establish model	• Cannot simulate physiological flow and shear stress	
Hollow-fiber<br>(1/19 studies)	*EC:*<br>hCMEC/D3 [b]<br>*Astrocytes:*<br>Primary	• Can mimic shear stress and physiological flow conditions<br>• Long term cell culture<br>• Easy to recover cell samples<br>• Versatile<br>• Easy to reconfigure<br>• Same platform device can be used for experiments of varying complexity<br>• Cylindrical – no sidewalls, no leaky edges<br>• Allows non-invasive observation<br>• Fiber thickness more closely mimics in vivo thickness of vessel walls	• Can be difficult to extract cell samples in some device designs<br>• More commonly support 2D cell cultures	[16]
Filter-free<br>(1/19 studies)	EC:<br>hCMEC/D3 [b]	• Better cell-cell interactions<br>• More physiologically relevant	• Limited working distance of high magnification microscopy limits image acquisition due to >2 mm thickness of collagen gel and use of conventional well plate	[15]
Hydrogel scaffold<br>(1/19 studies)	*EC:*<br>iPSC-BMEC [c]	• Hydrogels mimic many aspects of the natural extracellular matrix<br>• Observe cell behavior in a more physiology mimicking, 3D environment	• Channel sizes are still larger than in vivo vessel diameters	[35]

[a] Primary cell line; [b] Immortalized cell line; [c] Stem cell-derived cell line; [d] Cell line later identified to be a human urinary bladder carcinoma cell line, presenting many EC phenotypic characteristics [30]. BMEC, brain microvascular endothelial cell; DIPG, diffuse intrinsic pontine glioma; EC, endothelial cell; HA/hAst/HASTR, human astrocyte; HBEC/HBMEC/HUBEC/hCMEC, human brain/cerebral (microvascular) endothelial cell; HBPC/HBPCT, human brain pericyte; iBMEC, induced brain microvascular endothelial cell; iPSC, induced pluripotent stem cell.

Table S4. TEER values for transwell in vitro BBB/BBTB models.

TEER Method	Cell Types	TEER Values (Ω/cm²)	Refs
EVOM2 with Endohm-6 chamber electrode	iPSC-hBEC	458 ± 225	[24]
EVOM2, not specified	iPS-EC1	773 ± 52	[25]
	iPS-EC1 + astrocytes + pericytes + neurons	1267 ± 68	
	iPS-EC2	52 ± 3	
	iPS-EC2 + astrocytes + pericytes + neurons	150 ± 3	
	hCMEC/D3	45 ± 2	
	hCMEC/D3 + astrocytes + pericytes + neurons	67 ± 5	
EVOM2 with Endohm-6 chamber electrode	iBMEC (EC medium)	1423 ± 592	[23]
	iBMEC (neuron medium)	1920 ± 774	
	iBMEC + astrocytes + pericytes + neurons (EC medium)	1454 ± 263	
	iBMEC + astrocytes + pericytes + neurons (neuron medium)	1908 ± 582	
Millicell ERS-2 with STX01 electrode	HBMEC/ci18 EC	78.8 ± 4.2	[19]
	HBMEC/ci18 EC + HBPC/ci37 pericyte + HASTR/ci35 astrocyte	134.4 ± 5.5	
EVOM with Endohm-12 chamber electrode	ECV304 [a]	41.5 ± 2.12	[27]
	ECV304 + C6 rat glioma	25% decrease	
EVOM with STX2 electrode	HBEC-5i	35.8 ± 2.14	[29]
	HBEC-5i + HASTR media	39.8 ± 0.81	
EVOM, not specified	hCMEC/D3	32.9 ± 7.2	[28]
	hCMEC/D3 + U87 glioma	18.2 ± 6.7	

[a] ECV304 was later identified to be a human urinary bladder carcinoma cell line, presenting many EC phenotypic characteristics [30]. Data is expressed as mean ± SD for ≥3 independent experiments. BMEC, brain microvascular endothelial cell; EC, endothelial cell; EVOM, epithelial voltohmmeter; HASTR, human astrocyte; HBEC/HBMEC/hCMEC, human brain/cerebral (microvascular) endothelial cell; HBPC, human brain pericyte; iBMEC, induced brain microvascular endothelial cell; iPSC, induced pluripotent stem cell.

Table S5. Junctional protein and efflux transporter expression in in vitro BBB/BBTB models.

Model	Cell Type	Tight junction	Adherens junction	Transporters and other	Refs
Transwell	HUBEC [a]	Claudin-5<br>Occludin<br>ZO-1		P-gp<br>MRP2<br>OATP1	[32]
	HUBEC [a]<br>Glioma [a]	Claudin-5<br>Occludin<br>ZO-1		P-gp<br>MRP2<br>OATP1	
	hBMEC [a]	Claudin-5<br>ZO-1	CD31	vWF	[25]
	hCMEC/D3 [b]	Claudin-5<br>ZO-1	CD31	vWF	
	iPS-EC1 [c]<br>iPS-EC2 [c]<br>iPS-astrocyte [c]<br>iPS-pericyte [c]	Claudin-5<br>Occludin<br>ZO-1	CD31<br>VE-cadherin	vWF<br>caveolin1<br>GLUT1<br>P-gp<br>BCRP	
	ECV304 [b,d]	Claudin-5<br>Occludin<br>ZO-1			[27]
	ECV304 [b,d]	Claudin-5<br>Occludin<br>ZO-1			[30]
	HBEC-5i [b]	Claudin-5<br>ZO-1			[29]
	hCMEC/D3 [b]	Claudin-5<br>Occludin	VE-cadherin		[28]
	hCMEC/D3 [b]<br>U87 glioma [b]	Claudin-5<br>Occludin	VE-cadherin		
	HBMEC [a]	Claudin-5	VE-cadherin	P-gp<br>BCRP<br>GLUT1	[19]
	HBMEC/ciβ [b]	Claudin-5	VE-cadherin	P-gp<br>BCRP<br>GLUT1	
	HBMEC/ci18 [b]	Claudin-5<br>Occludin<br>ZO-1	VE-cadherin<br>CD31	vWF<br>P-gp<br>BCRP<br>LRP1<br>INSR<br>MRP4<br>GLUT1<br>MFSD2A<br>MCT8<br>TfR	
	HBMEC/ci18 [b] HBPC/ci37 [b] HASTR/ci35 [b]	Claudin-5<br>Occludin<br>ZO-1	VE-cadherin<br>β-catenin	P-gp<br>BCRP	
	hPSC-BMEC [c]	Claudin-5<br>Occludin<br>ZO-1		GLUT1<br>P-gp	[22]
	iBMEC [c]	Claudin-5<br>Occludin<br>ZO-1	VE-cadherin	vWF<br>Ulex<br>GLUT1<br>P-gp<br>LAT1<br>INSR<br>BCRP<br>MRP1	[23]
	iBMEC [c]<br>iCell Astrocyte [c]<br>Pericytes [a]<br>iCell GABANeuron [c]	Claudin-5<br>Occludin<br>ZO-1	VE-cadherin	vWF<br>Agglutinin-I<br>GLUT1<br>P-gp<br>LAT1	

				INSR<br>BCRP<br>MRP1	
	hBEC [a]	ZO-1	CD31<br>β-catenin	P-gp<br>LRP1<br>MRP1<br>BCRP<br>caveolin1<br>caveolin2<br>TfR<br>ISNR	[24]
	iPSC-hBEC [c]	Claudin-5<br>ZO-1	CD31	P-gp<br>LRP1<br>MRP1<br>BCRP<br>caveolin1<br>caveolin2<br>TfR<br>ISNR	
	CD34^{+}-EC [c]	Claudin-5<br>ZO-1		P-gp<br>BCRP<br>MRP1<br>MRP2	[33]
	CD34^{+}-EC [c]<br>DIPG-007 [a]<br>DIPG-013 [a]<br>DIPG-014 [a]	Claudin-5<br>ZO-1		P-gp<br>BCRP<br>MRP1<br>MRP2	
Microfluidic	hCMEC/D3 [b]	Claudin-5<br>ZO-1			[18]
	TY10 [b] (static)	Claudin-5	CD31<br>VE-cadherin	TfR	[34]
	TY10 [b] (perfused)	Claudin-5	CD31<br>VE-cadherin		
	HBMEC [b]	Occludin<br>ZO-1	CD31<br>VE-cadherin	P-gp<br>GLUT1<br>CERP<br>LRP1	[37]
	HBMEC [b]<br>Astrocytes [a]<br>Pericytes [a]	Occludin<br>ZO-1	CD31<br>VE-cadherin	P-gp<br>GLUT1<br>CERP<br>LRP1	
	iPSC-EC [c]	Claudin-5<br>Occludin<br>ZO-1		Laminin<br>Collagen IV<br>GLUT1<br>CERP<br>MRP1<br>MRP4<br>LAT1<br>LRP1<br>TfR<br>CAT1<br>MCT1<br>P-gp	[17]
	iPSC-EC [c]<br>Pericytes [a]	Claudin-5<br>Occludin<br>ZO-1		Laminin<br>Collagen IV<br>GLUT1<br>CERP<br>MRP1<br>MRP4<br>LAT1<br>LRP1<br>TfR<br>CAT1<br>MCT1<br>P-gp	

	iPSC-EC [c]<br>Astrocytes [a]<br>Pericytes [a]	Claudin-5<br>Occludin<br>ZO-1		Laminin<br>Collagen IV<br>GLUT1<br>CERP<br>MRP1<br>MRP4<br>LAT1<br>LRP1<br>TfR<br>CAT1<br>MCT1<br>P-gp	
Spheroidal	HBMEC [a]		CD31		[21]
	HBMEC [a]<br>Astrocytes [a]<br>HBVP [b]	Claudin-5<br>Occludin<br>ZO-1		P-gp<br>LRP1	
	hCMEC/D3 [b]		VE-cadherin<br>CD31	vWF	
	hCMEC/D3 [b]<br>Astrocytes [a]<br>HBVP [b]	Claudin-5<br>Occludin<br>ZO-1		P-gp<br>LRP1	
Hollow-fiber	hCMEC/D3 [b]	ZO-1		P-gp	[16]
Filter-free	hCMEC/D3 [b]	ZO-1			[15]
Hydrogel scaffold	iPSC-BMEC [c] (static)	Claudin-5<br>Occludin	VE-cadherin	MFSD2A<br>Caveolin1<br>GLUT1	[35]
	iPSC-BMEC [c] (perfused)	Claudin-5	VE-cadherin		
	HUVEC [a] (static)	Claudin-5	VE-cadherin		
	HUVEC [a] (perfused)	Claudin-5	VE-cadherin		
	μVas [a] (static)	Claudin-5	VE-cadherin	MFSD2A<br>Caveolin1	
	μVas [a] (perfused)	Claudin-5	VE-cadherin		

[a] Primary cell line; [b] Immortalized cell line; [c] Stem cell-derived cell line; [d] Cell line later identified to be a human urinary bladder carcinoma cell line, presenting many EC phenotypic characteristics [30]. μVas, microvascular; BCRP, breast cancer resistance protein; BMEC, brain microvascular endothelial cell; CAT1, cationic amino acid transporter 1; CERP, cholesterol efflux regulatory protein; DIPG, diffuse intrinsic pontine glioma; EC, endothelial cell; GLUT1, glucose transporter 1; HA/hAst/HASTR, human astrocyte; HBEC/HBMEC/HUBEC/hCMEC, human brain/cerebral (microvascular) endothelial cell; HBPC/HBPCT, human brain pericyte; HUVEC, human umbilical vein endothelial cell; iBMEC, induced brain microvascular endothelial cell; INSR, insulin receptor; iPSC, induced pluripotent stem cell; LAT1, L-type / large neutral amino acid transporter 1; LRP1, low-density lipoprotein receptor-related protein 1; MCT, monocarboxylate transporter; MRP, multi-drug resistance protein; P-gp, P-glycoprotein protein; OATP1, organic anion transporter polypeptide 1; TfR, transferrin receptor; VE-cadherin, vascular endothelial cadherin; vWF, von Willebrand factor; ZO-1, zonulae occludens-1.

Table S6. Permeability coefficients for in vitro BBB/BBTB models.

Cell Type	Compound	Molecular Weight (Da)	Permeability Coefficient (cm/s)	Refs
Fluorescent tracer				
hCMEC/D3	FITC-dextran	10,000<br>40,000	15×10^{-6}<br>3.7×10^{-6}	[18]
hCMEC/D3	Sodium salt<br>FITC-dextran	376<br>70,000	$5.99 \pm 4.91 \times 10^{-6}$<br>$4.95 \pm 2.37 \times 10^{-7}$	[36]
hCMEC/D3	FITC-dextran<br>Day 7	4000<br>40,000<br>70,000	$11.4 \pm 0.4 \times 10^{-6}$<br>$5.2 \pm 0.9 \times 10^{-6}$<br>$0.6 \pm 0.1 \times 10^{-6}$	[28]
hCMEC/D3 + U87		4000<br>70,000	$6.6 \pm 0.3 \times 10^{-6}$<br>$1.8 \pm 0.2 \times 10^{-6}$	
hCMEC/D3	FITC-dextran	4000	$1.33 \pm 0.012 \times 10^{-5}$	[31]
hCMEC/D3 (cAMP + rolipram treatment)		4000	$7.55 \pm 0.005 \times 10^{-6}$	
hCMEC/D3 (arachidonic acid treatment)		4000	$3.17 \pm 0.064 \times 10^{-5}$	
hCMEC/D3<br>(hollow-fiber)	FITC -Dextran<br>Day 7<br>Day 14	4000	$8.33 \pm 0.007 \times 10^{-6}$<br>$3.33 \pm 0.001 \times 10^{-6}$	[16]
hCMEC/D3 (filter-free)	FITC-dextran	4000<br>2,000,000	$6.17 \pm 0.004 \times 10^{-6}$<br>$4.50 \pm 0.000 \times 10^{-7}$	[15]
hCMEC/D3 (transwell)		4000<br>2,000,000	$5.27 \pm 0.003 \times 10^{-6}$<br>$3.17 \pm 0.000 \times 10^{-7}$	
HBEC−5i ECs + EC medium	Na-Fl<br>FITC-dextran	376<br>4000	$7.8 \pm 0.1 \times 10^{-6}$<br>$6.4 \pm 0.2 \times 10^{-6}$	[29]
HBEC-5i ECs + HA medium	Caffeine<br>Na-Fl<br>FITC-dextran	212<br>376<br>4000	$67.0 \pm 4.4 \times 10^{-6}$<br>$5.7 \pm 0.1 \times 10^{-6}$<br>$3.6 \pm 0.1 \times 10^{-6}$	
iPSC-ECs	FITC-dextran	10,000<br>40,000	12×10^{-7}<br>6.6×10^{-7}	[17]
iPSC-ECs + human primary pericytes		10,000<br>40,000	4.8×10^{-7}<br>2.5×10^{-7}	
iPSC-ECs + human primary astrocytes + human primary pericytes		10,000<br>40,000	2.2×10^{-7}<br>8.9×10^{-8}	
iPSC-derived BMEC	FITC-dextran Day 1	3000	$1.2 \pm 0.6 \times 10^{-7}$ [b]<br>$1.9 \pm 0.2 \times 10^{-7}$ [c]	[35]
	FITC-dextran Day 7	3000	$4.5 \pm 2 \times 10^{-7}$ [b]<br>$1.4 \pm 0.8 \times 10^{-7}$ [c]	
	FITC-dextran Day 14	3000	$20.1 \pm 26 \times 10^{-7}$ [b]<br>$0.4 \pm 0.3 \times 10^{-7}$ [c]	
HUVEC	FITC-dextran Day 1	3000	$118 \pm 28 \times 10^{-7}$ [b]<br>$119 \pm 130 \times 10^{-7}$ [c]	
	FITC-dextran Day 7	3000	$69.8 \pm 10 \times 10^{-7}$ [b]<br>$76.2 \pm 49 \times 10^{-7}$ [c]	
	FITC-dextran Day 14	3000	$195 \pm 150 \times 10^{-7}$ [b]<br>$228 \pm 48 \times 10^{-7}$ [c]	
μVas	FITC-dextran Day 1	3000	$5 \pm 3.6 \times 10^{-7}$ [b]<br>$6 \pm 2.3 \times 10^{-7}$ [c]	
	FITC-dextran Day 7	3000	$31 \pm 3 \times 10^{-7}$ [b]<br>$5.3 \pm 1 \times 10^{-7}$ [c]	
	FITC-dextran Day 14	3000	$33 \pm 0.9 \times 10^{-7}$ [b]<br>$15.6 \pm 13.7 \times 10^{-7}$ [c]	
Blank inserts	Lucifer Yellow	452	$1.0 \pm 0.001 \times 10^{-5}$	[30]

ECV304			5.17 ± 0.000 × 10^{-6}	
CD34⁺-EC + pericytes	Lucifer Yellow	452	9.0 ± 0.001 × 10^{-6}	[33]
CD34⁺-EC + pericytes + astrocytes	Day 1		1.13 ± 0.001 × 10^{-5}	
	Day 7		1.37 ± 0.002 × 10^{-5}	
CD34⁺-EC + pericytes + DIPG-007	Day 1		1.15 ± 0.001 × 10^{-5}	
	Day 7		1.25 ± 0.001 × 10^{-5}	
CD34⁺-EC + pericytes + DIPG-013	Day 1		1.25 ± 0.001 × 10^{-5}	
	Day 7		1.22 ± 0.000 × 10^{-5}	
CD34⁺-EC + pericytes + DIPG-014	Day 1		1.22 ± 0.001 × 10^{-5}	
	Day 7		1.30 ± 0.001 × 10^{-5}	
hBMEC/ci18	Na-Fl	376	32 ± 4 × 10^{-6}	[19]
	Lucifer Yellow	452	30 ± 4 × 10^{-6}	
	Rhodamine123	381	9 ± 1 × 10^{-6}	
hBMEC/ci18 + HASTR/ci35 astrocytes + HBPC/ci37 pericytes	Na-Fl	376	18 ± 4 × 10^{-6}	
	Lucifer Yellow	452	18 ± 4 × 10^{-6}	
	Rhodamine123	381	5 ± 4 × 10^{-6}	
ECV304 [a]	Rhodamine123	381	12.38 ± 0.91 × 10^{-6}	[27]
Chemicals (not included in Figure 6)				
hCMEC/D3	Urea	60	2.96 ± 0.11 × 10^{-5}	[26]
	Mannitol	182	1.98 ± 0.05 × 10^{-5}	
	Sucrose	342	1.52 ± 0.13 × 10^{-5}	
	Inulin	5000	8.46 ± 0.02 × 10^{-6}	
	PEG-4000	4000	3.93 ± 0.36 × 10^{-6}	
hCMEC/D3 + primary astrocytes (direct co-culture)	Urea	60	2.43 ± 0.15 × 10^{-5}	
	Mannitol	182	1.52 ± 0.07 × 10^{-5}	
	Sucrose	342	1.17 ± 0.008 × 10^{-5}	
	Inulin	5000	7.55 ± 0.3 × 10^{-6}	
	PEG-4000	4000	3.57 ± 0.10 × 10^{-6}	
hCMEC/D3 + primary astrocytes (indirect co-culture)	Mannitol	182	1.89 ± 0.15 × 10^{-5}	
	Sucrose	342	1.53 ± 0.12 × 10^{-5}	
iPSC-hBEC	Propranolol	259	21.7 ± 3.1 × 10^{-6}	[24]
	Sucrose	342	2.9 ± 1.6 × 10^{-6}	
Drugs				
ECV304 [a]	Propranolol	259	28.42 ± 1.25 × 10^{-6}	[30]
	Verapamil	455	23.25 ± 0.87 × 10^{-6}	
	Quinidine	324	24.46 ± 1.61 × 10^{-6}	
	Digoxin	781	3.29 ± 0.16 × 10^{-6}	
hBMEC/ci18	Propanolol	259	1872 ± 749 × 10^{-6}	[19]
	Pyrilamine	285	854 ± 218 × 10^{-6}	
	Memantine	179	849 ± 233 × 10^{-6}	
	Dipenhydramine	255	681 ± 195 × 10^{-6}	
	Quinidine	324	501 ± 224 × 10^{-6}	
	Dantrolene	314	199 ± 50 × 10^{-6}	
	Desloratadine	311	301 ± 105 × 10^{-6}	
hBMEC/ci18 + HASTR/ci 35 astrocytes + HBPC/ci37 pericytes	Propanolol	259	1280 ± 686 × 10^{-6}	
	Pyrilamine	285	1398 ± 324 × 10^{-6}	
	Memantine	179	640 ± 122 × 10^{-6}	
	Dipenhydramine	255	523 ± 100 × 10^{-6}	
	Quinidine	324	161 ± 31 × 10^{-6}	
	Dantrolene	314	163 ± 11 × 10^{-6}	
	Desloratadine	311	72 ± 60 × 10^{-6}	
hPSC-BMEC	Atenolol	226	4.64 ± 0.38 × 10^{-6}	[22]
	Cimetidine	252	7.84 ± 0.38 × 10^{-6}	
	Prazosin	420	10.36 ± 0.07 × 10^{-6}	
	Hydroxyzine	448	16.36 ± 3.33 × 10^{-6}	
	Caffeine	212	119.4 ± 34.6 × 10^{-6}	
	Donepezil	433	40.5 ± 3.00 × 10^{-6}	
	Memantine	216	43.0 ± 2.41 × 10^{-6}	

	Rivastigmine	400	80.7 ± 9.39 × 10^{-6}	
	IgG	150,000	2.99 ± 0.64 x 10^{-9}	
iPS-EC1	Atenolol	226	10.5 ± 3.1 × 10^{-6}	[25]
	Erythromycin	734	11.6 ± 3.0 × 10^{-6}	
	Verapamil	455	12.5 ± 0.9 × 10^{-6}	
	Dantrolene	314	22.1 ± 2.3 × 10^{-6}	
	Phenytoin	252	25.7 ± 1.6 × 10^{-6}	
	Propranolol	259	25.1 ± 4.9 × 10^{-6}	
iPS-EC1 + astrocytes + pericytes + neurons	Atenolol	226	4.7 ± 1.0 × 10^{-6}	
	Erythromycin	734	9.9 ± 2.5 × 10^{-6}	
	Verapamil	455	11.2 ± 4.0 × 10^{-6}	
	Dantrolene	314	15.2 ± 3.1 × 10^{-6}	
	Phenytoin	252	35.6 ± 3.4 × 10^{-6}	
	Propranolol	259	22.6 ± 5.5 × 10^{-6}	
iPS-EC2	Atenolol	226	30.4 ± 0.9 × 10^{-6}	
	Erythromycin	734	21.5 ± 3.0 × 10^{-6}	
	Verapamil	455	22.3 ± 0.9 × 10^{-6}	
	Dantrolene	314	41.3 ± 5.8 × 10^{-6}	
	Phenytoin	252	29.2 ± 5.2 × 10^{-6}	
	Propranolol	259	31.8 ± 3.5 × 10^{-6}	
iPS-EC2 + astrocytes + pericytes + neurons	Atenolol	226	34.6 ± 8.1 × 10^{-6}	
	Erythromycin	734	31.8 ± 6.2 × 10^{-6}	
	Verapamil	455	18. 3 ± 2.8 × 10^{-6}	
	Dantrolene	314	45.6 ± 3.7 × 10^{-6}	
	Phenytoin	252	29.4 ± 10.9 × 10^{-6}	
	Propranolol	259	10.7 ± 1.2 × 10^{-6}	
Chemotherapy				
CD34⁺-ECs + astrocytes	TMZ	194	8.33 ± 0.000 × 10^{-6}	[33]
	Panobinostat	349	5.50 ± 0.000 × 10^{-6}	
CD34⁺-EC + DIPG-007	TMZ	194	7.17 ± 0.000 × 10^{-6}	
	Panobinostat	349	7.83 ± 0.001 × 10^{-6}	
CD34⁺-EC + DIPG-013	TMZ	194	7.67 ± 0.000 × 10^{-6}	
	Panobinostat	349	4.83 ± 0.000 × 10^{-6}	
CD34⁺-EC + DIPG-014	TMZ	194	8.33 ± 0.000 × 10^{-6}	
	Panobinostat	349	4.00 ± 0.000 × 10^{-6}	
Antibodies				
TY10 + hAst astrocytes + hBPCT pericytes	Anti-TfR (MEM-189), IgG1	95,000	4.83 × 10^{-7}	[34]
	Anti-hen egg lysozyme, IgG1	93,000	2.67 × 10^{-7}	

[a] Cell line later identified to be a human urinary bladder carcinoma cell line, presenting many EC phenotypic characteristics [30]. Performed under [b] static or [c] perfused conditions. For studies reporting permeability coefficients in cm/min these were converted to cm/sec to enable easy comparison. Data is expressed mean ± SD for the reported number of experiments. μVas, microvascular; BMEC, brain microvascular endothelial cell; cAMP, cyclic adenosine monophosphate; DIPG, diffuse intrinsic pontine glioma; EC, endothelial cell; EVOM, epithelial voltohmmeter; FITC, fluorescein isothiocyanate; HA/hAst/HASTR, human astrocyte; HBEC/HBMEC/hCMEC, human brain/cerebral (microvascular) endothelial cell; HBPC/hBPCT, human brain pericyte; HUVEC, human umbilical vein endothelial cell; iBMEC, induced brain microvascular endothelial cell; iPSC, induced pluripotent stem cell; Na-Fl, sodium fluorescein; TMZ, temozolomide.

© 2021 by the authors. Licensee MDPI, Basel, Switzerland. This article is an open access article distributed under the terms and conditions of the Creative Commons Attribution (CC BY) license (http://creativecommons.org/licenses/by/4.0/).
